# Modulation of Gut Microbiota by Essential Oils and Inorganic Nanoparticles: Impact in Nutrition and Health

**DOI:** 10.3389/fnut.2022.920413

**Published:** 2022-07-08

**Authors:** Veronica Lazar, Alina-Maria Holban, Carmen Curutiu, Lia Mara Ditu

**Affiliations:** ^1^Department of Botany and Microbiology, Faculty of Biology, University of Bucharest, Bucharest, Romania; ^2^Laboratory of Microbiology, Research Institute of the University of Bucharest, Bucharest, Romania

**Keywords:** microbiota interference, dysbiosis, biofilms, essential oils, bioactive nanoparticles

## Abstract

Microbiota plays a crucial role in human health and disease; therefore, the modulation of this complex and yet widely unexplored ecosystem is a biomedical priority. Numerous antibacterial alternatives have been developed in recent years, imposed by the huge problem of antibioresistance, but also by the people demand for natural therapeutical products without side effects, as dysbiosis, cyto/hepatotoxicity. Current studies are focusing mainly in the development of nanoparticles (NPs) functionalized with herbal and fruit essential oils (EOs) to fight resistant pathogens. This is due to their increased efficiency against susceptible, multidrug resistant and biofilm embedded microorganisms. They are also studied because of their versatile properties, size and possibility to ensure a targeted administration and a controlled release of bioactive substances. Accordingly, an increasing number of studies addressing the effects of functional nanoparticles and plant products on microbial pathogens has been observed. Regardless the beneficial role of EOs and NPs in the treatment of infectious diseases, concerns regarding their potential activity against human microbiota raised constantly in recent years. The main focus of current research is on gut microbiota (GM) due to well documented metabolic and immunological functions of gut microbes. Moreover, GM is constantly exposed to micro- and nano-particles, but also plant products (including EOs). Because of the great diversity of both microbiota and chemical antimicrobial alternatives (i.e., nanomaterials and EOs), here we limit our discussion on the interactions of gut microbiota, inorganic NPs and EOs. Impact of accidental exposure caused by ingestion of day care products, foods, atmospheric particles and drugs containing nanoparticles and/or fruit EOs on gut dysbiosis and associated diseases is also dissected in this paper. Current models developed to investigate mechanisms of dysbiosis after exposure to NPs/EOs and perspectives for identifying factors driving EOs functionalized NPs dysbiosis are reviewed.

## Introduction

The wide assortment of microorganisms inhabiting the mammalian gastrointestinal tract, named gut microbiota (GM), has a central position in health and disease. Human microbiota contains more than 100 trillion symbiotic microorganisms inhabiting on and within their host body, being considered as “essential organ” (1). Microbiota species carry approximately 150 times more genes than are found in the entire human genome, and the collection of genomes from all these microorganisms is known as the microbiome (2).

Despite its wide diversity, most GM bacterial species belong to the following major phyla: Actinobacteria, Bacteroidetes, Firmicutes, and Proteobacteria. The gastrointestinal (GI) tract colonization starts during birth, recent evidence showing that the placenta may harbor microorganisms, including pathogenic ones (3–5). Depending on the delivery mode, initial microbial communities tend toward a skin-like (cesarean section) or a vaginal-like (vaginal delivery) configuration. Usually, first colonizers (pioneer bacteria) of a new-born belong to *Staphylococcus, Corynebacterium, Propionibacterium, Lactobacillus, Prevotella* and *Sneathia* genera (6). After 1 month of life, Enterobacteria species become dominant in the GI, but after 6 months microbiota switches toward a *Bifidobacterium* and *Bacteroides* predominant population. After the solid food is introduced, Bacteroides and Firmicutes become the dominant bacteria groups. At the same time, the immune system “learns” to differentiate between commensal and pathogenic bacteria. In adulthood, a specific segregation of microbiota species depending on the GI segments, could be distinguished, and these differences are strongly related to pH variations among the GI segments (4, 6, 7).

Microbiota controls normal host physiology, such as nutritional status, energy metabolism, immunity, infection, behavior and stress response, explaining its tolerance by the host organism (4). A well-balanced gut microbiota is essential to ensure homeostasis at the intestinal mucosa and beyond (5, 8).

Despite intense research was done in microbiota composition and physiology, the mechanisms responsible for its beneficial or detrimental influences on the host remain largely undefined. Joint analyses of high-throughput human multi-omics data, including metagenomics and metabolomics data, together with measures of host physiology and mechanistic experiments in humans, animals and cells reveal sophisticated signaling pathways between microbiota species and host (9). Generally, a wider microbiota diversity corelates with a health status, while lower bacterial diversity has been reproducibly observed in people with metabolic disorders, such as inflammatory bowel disease, psoriatic arthritis, type 1 diabetes, atopic eczema, coeliac disease, obesity, type 2 diabetes, and arterial stiffness (10, 11).

This dynamic microbial community varies widely from an individual to another and may change several times during the lifetime of an individual. Microbiota is susceptible to virtually any exogenous and endogenous changes, and the change of its pattern further impact on the host health status.

Molecular factors impacting on the GM can be clustered in three main categories: (i) host-derived factors, which include specific microRNAs, and nonspecific molecules, such as antimicrobial peptides, mucus, immunoglobulin A, and hormones (12); (ii) microbial factors, such as LPS (lipopolysaccharides), metabolites, SCFAs (short chain fatty acids), and QS (Quorum Sensing) signaling molecules (13); (iii) dietary molecules (i.e., fats, sugars, proteins); (iv) medications (mainly antibiotics, but any drugs could impact the gut microbiota, including natural products utilized in traditional medicine, prophylaxis or immune stimulation); and (v) various nano- and microstructured particles, which could reach the GI tract accidentally, by ingestion or by means of oral therapy (14).

Diet, but also molecules and particles which could be ingested in various situations, represent the most significant shaping agents for GM (6). Among these, essential oils (EOs) and plant extracts are frequently reaching microbiota following ingestion (15). Moreover, various micro and nanoparticles (NPs) widely encountered in the environment, dust, air, water but also medication and self-care products (mouthwashes, tooth cream, sunscreen etc.) could reach the gut microbiota, with significant consequences (16).

The purpose of this review is to discuss the main interactions in the microbiota-NPs-EOs triad, emphasizing on the role of EOs and NPs in shaping microbiota and modulate the gut metabolism, which could impact on nutrition and health/disease balance.

## Essential Oils (EOs) in Shaping Microbiota

Plant-derived medicines are an integral part of complementary and alternative medicine worldwide that are used for therapeutic purposes by up to 75% of the general population. The use of plant therapy against metabolic diseases in which microbiota plays an important role, such as obesity, is increasing (17). The potential of fruits, aromatic plants and spices for improving human health is primary owing to their EOs which harbor various biological activities (6). The main targets of fruit EOs therapy concerning to healthy gut functions in humans are GI infection, inflammation, metabolic diseases and carcinogenesis (18).

EOs are highly volatile metabolites of plants, obtained by various techniques, as steam distillation, hydro-distillation, or by organic-solvent extraction of the plant and are usually colorless and fluid at room temperature (6, 19). They generally contain aromatic compounds, as hydrocarbons and their oxygenated derivatives, each EO being a complex mixture of up to 200 different organic compounds, most of them present in low amounts (<1%) (20).

Bioactive compounds found in the fruit EOs could interact with the GM by various means, such as modification of the gut environment under microbial metabolization of the EO bioactives or by host excreted compounds. Such changes could impact on the microbiota composition and density but also exert more subtle changes, such as alteration of the microbiota ability to synthesize certain metabolites, important for the host (i.e., SCFAs and vitamins) (21, 22). These interferences would inevitably impact on the host health.

Polyphenols are secondary metabolites with over 10,000 structural variants, found in most fruits. These bioactive phytochemicals are divided into *flavonoids* (flavonols, flavanones, flavanols, flavones, isoflavones, and anthocyanidins) and *non-flavonoids* (phenolic acids, stilbenes, coumarins, xanthones, lignans, and curcuminoids). Polyphenols are abundantly consumed as antioxidants, in quantities of up to 1 g/day by Western population (18, 23). Flavanones, flavonols, isoflavone, anthocyanins, proanthocyanidins, and resveratrol are the most studied polyphenols with significant impact on the GI tract function, impacting on insulin signaling, downregulation of oxidative stress, gut bacteria modulation, improvement of endothelial dysfunction, modulation of intestinal absorption and metabolism (18, 24).

There are data showing that the microbial metabolism of polyphenols can influence their bioactivity; consequently, inter-individual variation in microbiota pattern and metabolism could have significance for the health benefits of phytochemicals in general (25). For instance, phytoestrogens are polyphenols similar to human estrogens found in plants or vegetal food products, such as soy beans, flaxseed and other seeds, cereals, vegetables, fruits, tea, chocolate etc. In the phytoestrogens class there are different chemical compounds (stilbenes, coumestans, isoflavones, ellagitannins, and lignans), with a similar structure to human, but which can exert both estrogenic and antiestrogenic effects. Isoflavones, ellagitanins, and lignans are metabolized by intestinal bacteria to produce equol, urolithins, and enterolignans, which are more bioavailable, and have more estrogenic/antiestrogenic and antioxidant activity by comparison with their precursors; furthermore, these metabolites have anti-inflammatory, antiproliferative and apoptosis-inducing effects (26, 27). The isoflavone daidzein extracted from soy seeds is metabolized by microbiota in equol (isoflavandiol–a non-steroidic estrogen) which is more bioactive than daidzein, regarding oestrogenic and anti-oestrogenic activity, antioxidant capacity and potential anti-cancer effects (26). Also, urolithins are microbial metabolites produced by the human GM from ellagitannins and ellagic acid (EA); these metabolites are better absorbed than their precursors and are considered responsible for the health effects attributed to ellagitannins and EA, present in food such as berries and nuts. It has been observed an interindividual variability concerning the production of urolithins (metabotypes) and its relationship with dysbiosis and host health status. The transformation of isoflavones, ellagitanins, and lignans by intestinal microbiota is essential for the protective effect against certain diseases, such as cancer, cardiovascular disease, osteoporosis, but also menopausal symptoms (26, 27).

Ursolic acid (UA), a natural pentacyclic triterpenoid, present in fruit peels (apples, prunes, bilberries, cranberries, hawthorn) and in many herbs and spices (rosemary, thyme, basil, oregano, pepermint, lavender), has been largely used as a phytomedicine, showing numerous pharmacological activities (28). UA and its synthetic derivatives proved anticancer, antidiabetic, antiarrhythmic, anti-hyperlipidemic and anti-hypercholesterolemic, antimicrobial, hepatoprotective and hepatoregenerative properties (29). In addition to its well-documented antiinflammatory effect, UA can reduce the neuronal damage in metabolic syndrome mice. Added to mice diet, UA is able to increase the mass muscle and brown fat, while reducing white fat, and thus obesity and correlated conditions (30).

### *In vivo* Models for Microbiota–EOs Interactions

Metabolomics analysis have been recently developed by utilizing complex omics platforms to investigate the effects of EO bioactives on microbiota and their host metabolism. However, specific interactions of fruit EOs-microbiota-host are difficult to investigate, since most of the analyzed biological samples are stool microbiota with free bacteria or blood samples, but not mucosal biopsies with adherent bacteria which should be ideally processed to cover the whole microbiota (31). Various animal models are currently investigated in order to monitor the microbiota-EOs interferences not only in feces, but also in the different regions of the gut (i.e., adhered to the GI mucosal proteins and epithelial cells) and outside the gut (i.e., central nervous system, systemic immune system, metabolites in the bloodstream etc.) (22).

Although rodents have been most widely used as model animals for studying *in vivo* interactions of gut microbiota with various molecules and particles, pigs are considered a more reliable alternative, since they display more anatomical, physiological and metabolic similarities to humans (22).

Studies showed that EOs could increase the weight gain and improve resistance to infection in swine and poultry (32). Besides, fruit EOs showed beneficial effects on antioxidant status, intestinal morphology, and barrier in animals (33).

Terpenes are a class of aromatic compounds found in EOs of many plants as well as fruit peels. These compounds define the distinctive smell of such fruits and plants as pine, citrus, thyme, cannabis, hops, etc. (34). Several studies show that terpenes display pharmacological and therapeutical activities such as anti-inflammatory, antioxidant, antibacterial and gastroprotective (35). The active component d-Limonene, which is present in the majority of the citrus family of fruits (such as sweet oranges, grapefruits and lemons) (36), improve metabolic parameters and modulate the intestinal microbiota.

An *in vivo* study on mouse model proved a significant decrease in weight gain which corelate with microbiota changes. Obese mice fed with d-Limonene based food supplements for 86 days showed lower cholesterolemia and reduced systemic inflammation parameters. These changes were compared with d-Limonene induced microbiota modulation, by decreasing the number of potentially pro-inflammatory taxa, such as *Desulfovibrionaceae* (known sulfate-reducing pathobionts), *Peptostreptococcaceae* and *Erysipelotrichaceae*, which are typically increased in metabolic inflammatory disorders, and increasing the number of *Bacillaceae, Planococcaceae* and *Clostridiaceae* bacteria. Thus, the study showed a positive modulation in gut microbiota and also demonstrated that even at higher doses, d-Limonene did not exert toxic manifestations (37). Similar effects were demonstrated in obese rat models, feed with microcapsules containing d-limonene extracted from sweet orange EOs. Dahu et al., demonstrated that d-limonene change the composition and abundance of microbiota at phylum- and genus-levels, correlated with body weight loss and improved fat metabolism (38).

Carvacrol is part of phenolic monoterpenes that poses pharmacological effects as anti-inflammatory, antioxidant, antibacterial and gastroprotective activity. Carvacrol was administered in rat models with intrarectal induced colitis to evaluate *in vivo* interference with the gut. After 3 days of treatment, it was observed a reduction in macroscopic and microscopic damage of the inner lining of the colon, and also an increase in antioxidant molecules and enzymes [catalase (CAT), superoxide dismutase (SOD) and glutathione peroxidase (GPx)]. These carvacrol-induced molecular changes at the gut level helped decrease the oxidative stress and could keep a balanced, healthy microbiota. The study concluded that the applied Carvacrol resulted in reducing inflammatory, nociceptive, and oxidative damage in rats (35)^.^

Previous studies found that carvacrol and thymol could reduce the populations of *Escherichia coli* and *Clostridium perfringens*, while increasing the number of lactobacilli cells in the intestine of broilers (15). However, the effects of such compounds on the intestinal microbiota and microbial metabolites are limited. Table 1 offer a recent overview regarding bioactive compounds from fruit EOs which were investigated for gut interference and microbiota modulation.

**Table 1:** Bioactive molecules from fruit EOs with gut and microbiota modulation activity and their study models.

**Fruit EOs**	**Active molecules**	**Study models**	**Microbiota modulation**	**Health impact**	**References**
Sweet orange EOs	d-limonene	*In vivo* studies on obese rats	Change in the composition and abundance of microbiota at phylum- and genus-levels	Body weight loss and improved fat metabolism	(38)
		*In vivo* studies in High-Fat Diet mice	Decrease the number of potentially pro-inflammatory microbiota taxa	Lower cholesterolemia, reduce systemic inflammation	(37)
Citronella EOs	6-octenal,3,7-dimethyl- citronellal	*In vitro* studies on bacteria cells	Antibacterial activity against *bacillus sp., e. Coli, listeria monocytogenes, proteus vulgaris, s. Aureus, candida albicans* and *aspergillus sp*.	Lower the number of opportunistic taxa	(6, 39)
Bergamot EOs	CarvacrolTerpene oxides	*In vitro* studies on bacteria cells	Impacts growth and survival in *s. Typhimurium* and *e. Coli*	Modulate cell physiology and expression of stress-response proteins	(6, 40)
Grapefruit EOs	Limonene Terpene oxides	*In vivo* studies on obese rats	Microbiota disruption at high concentrations	Increase lipolysis and suppressed body weight gain	(6, 41)
Lime EOs	Monoterpenes Terpenes	*In vivo* studies on obese rats	Microbiota disruption at high concentrations	Affects the food intake and energy expenditure which act as weight gain suppressant	(6, 42)
Tangerine EOs	d-Limonene γ-Terpinene	*In vitro* study on bacterial cells	Modulate growth of: *campylobacter jejuni, e. Coli, l. Monocytogenes* and *salmonella enterocolitica*	Anti-pathogenic activity in the gut	(43)
Lemon EOs	d-Limoneneβ-Pinene	*In vivo* study on rat models	Microbiota disruption at high concentrations	Increase sympathetic nerve activity to white adipose tissue metabolism, increase lipolysis and suppressed body weight gain	(6, 44)
Bitter Melon EOs	β-sitosterol	*In vivo* study on rat models	Unknown microbiota changes	Increased fatty acid oxidation, decreased adipose leptin levels, reduce white adipose tissue and visceral fat	(6, 45)
Apple and grape EOs	(+) catechin	*In vitro* on fecal bacteria	Growth stimulation of *clostridium coccoides – eubacterium rectale, bifidobacterium spp.*, and *escherichia coli*, growth inhibition of *clostridium histolyticum*	Anti-pathogenic activity in the gut	(46, 47)
	(-) epicatechin	*In vitro* on fecal bacteria	Growth stimulation of *c. Coccoides – e. Rectale*.	Anti-pathogenic activity in the gut	(46, 47)
Grapes EOs	Resveratrol	*In vivo*(Randomized,double-blind,placebo- controlled clinical trial)	Increase absolute abundances of γ-proteobacteria and bacteroidetes phyla, while reducing firmicutes and actinobacteria abundance in obese and overweight men and woman	Increase fat oxidation and change the overall gut metabolism	(48)

## Nanoparticles (NPs)–Gut Microbiota Interference

NPs are intensively investigated in recent years for the development of numerous industries, such as food, medicine, pharmacology, construction, cosmetology and many more. The advantages of the NPs in the biomedical field are clearly discernible, recent studies describing the progress made by NPs research in infection control (49) and cancer (50), empathizing on their highly controllable targeting and organ specific interactions (51, 52). However, little is known regarding their mid- and long-term side effects or the impact of these nanosized materials on environmental or mammalian microbiota (16).

It is estimated that we are daily exposed to the ingestion of more than 10^13^ micron or nanosized persistent particles which inevitably reach our gut microbiota (14). NPs are widely found in our actual environment, daily care products, foods, and numerous pharmaceutical products (16). Inorganic NPs, such as silver (Ag), silicon dioxide (SiO_2_), titanium dioxide (TiO_2_), and zinc oxide (ZnO) are most relevant for ingestion because they are added as ingredients or preservatives to foods and supplements and are also contained by routinely utilized healthcare products, such as toothpaste, mouthwash, sunscreens, gels, capsules etc. (14). TiO_2_, Ag and SiO_2_ are commonly used as food coloring and anti-caking agents but also in the design of (antimicrobial) active food packaging, while ZnO is used as food supplement (given that zinc is an essential trace element) (53).

From a biomedical perspective, NPs are recognized as efficient antimicrobial agents, able to interfere with growth, virulence features and also viability of pathogenic bacteria in a dose, strain and NP type-specific manner. The antibacterial mechanisms are yet to be fully described; however, recent scientific progress proposed some interactions responsible for their effects. These are: (i) indirect interference with biological activity by release of metal ions from the metal nanoparticles, (ii) direct interaction of the metal ions with intracellular components, (iii) direct interaction of NPs with the cell wall through electrostatic interactions, leading to impaired membrane function and impaired nutrient assimilation; (iv) formation of extracellular and intracellular reactive oxygen species (ROS), and damage of lipids, proteins and DNA by oxidative stress; (v) high-levels of metal-binding to the cell envelope and high ROS levels causing damage to the plasma membrane and leakage of the cell content; (vi) direct NPs interactions with proteins and DNA, impairing their function and disturbing the cellular metabolism in addition to, vii) targeted release of bioactive drugs or molecules (such as antibiotics, biocides, plant derived compounds, EOs) contained into the nanosystems (54–56).

Antimicrobial activity of the NPs is widely manifested against virtually any type of microorganisms–bacteria, yeast, microfungi, protozoa, but also viruses, regardless their virulence, antibiotic resistance and ability to develop biofilms (49, 57). Due to the wide antimicrobial properties of NPs and their presence in numerous products which facilitate gut access, their interactions with microbiota started to be intensively investigated in recent years. Multiple *in vitr*o and *in vivo* mammalian models have been utilized to reveal the impact of NPs on the gut microbiota and the role of this interaction in balancing health and disease. Given the very limited information on nanostructured food, cosmetic and even pharmaceutical additives, most research was done with NP models.

### Silver (Ag) NPs

AgNPs are used in hundreds of commercial products and studies demonstrated their microbiota modulatory potential *in vitro* and *in vivo*.

NPs can penetrate into human body by ingestion, inhalation, or dermal contact, the dietary ingestion being considered the primary penetrance route of silver nanoparticles (AgNPs) (58).

*In vivo* studies were conducted in rats or mice using high concentrations (up to 36 mg/kg bw/d), as compared to human dietary levels (0.03 to 0.65 μg/kg bw/d). Recent studies reported contradictory results regarding microbiota modulation, most researchers concluding that AgNPs increase the proportions of *Bacteroidetes* and pathogenic gram-negative bacteria, while decreasing the proportions of *Firmicutes, Lactobacillus* and *Bifidobacterium* in the mammalian gut (59). AgNPs derived microbiota alterations depend on the shapes of NPs, suggesting that their structure (which affects the surface-to-volume ratio) could determine the reactivity with gut bacteria. Moreover, the shift in the Firmicutes/Bacteroidetes ratio has been confirmed in the fecal microbiota of rats and mice orally given Ag-NPs at 2.5 or 3.6 mg/kg bw/d for 7–14 days (60). There are also reports about different effects of AgNPs on gut microbiota, depending on their shape such as cubes and spheres; for instance, AgNPs as cubes decreased *Clostridium spp., Bacteroides uniformis, Christensenellaceae*, and *Coprococcus eutactus* whereas, NPs as spheres reduced the relative abundance of *Oscillospira spp., Dehalobacterium spp., Peptococcaeceae, Corynebacterium spp., Aggregatibacter pneumotropica* (61).

In a study of Lyu and coworkers (2021) (62) performed on mice, the results showed that relative abundance of several bacteria was greater in the exposed group to AgNP, by comparison with the control group. These abundant bacteria belong to *Prevotella spp., Bacillus spp., Staphylococcus spp., Enterococcus spp*., *Ruminococcus* spp. and, *Planococcaceae*. Other few bacteria were less abundant in mice developmentally exposed to AgNPs, such as *Coprobacillus spp., Mucispirillum spp*., and *Bifidobacterium* spp. Or, it is demonstrated that in humans an increased relative abundance of *Prevotella spp*. is correlated with greater body mass index (BMI) (63), with colitis and an increased intestinal inflammation (64).

Exposure to low amounts of AgNPs induce *in vivo* toxic symptoms in *Daphnia magna*, which are corelated with diversity changes in gut microbiota. AgNPs caused concentration dependent down-regulation of genes encoding proteasome, ATP synthesis, fatty acid biosynthesis, and β-oxidation in the host when utilized in lower amounts, while higher NPs concentrations showed a more pronounced gene-regulation effect, ed with shorter body length, decreased digestion and reproduction ability in exposed *D. magna*. Microbial sulfidation of Ag^+^ ions, *via* the aggregation of nanoparticles and possibly the recovered synthesis of SCFAs relieved the observed AgNPs induced changes in the host. Symptomatic palliation observed in the AgNPs-tolerant microbiota inoculated *D. magna* further confirmed the detoxifying role of gut microbiota (65).

The impact of AgNPs on human microbiota is less investigated. *In vitro* study was done to investigate the short-term impacts of Ag-NPs on a defined human bacterial community called microbial ecosystem therapeutic-1 (MET-1, consisting of 33 bacterial strains, established from stool obtained from a healthy donor). Exposure to high concentrations of AgNPs (1–200 mg/L) for 48 h resulted in culture-generated gas production and changes in fatty acid methyl ester profiles. Bacterial composition modulation was characterized by decreased abundances of *Bacteroides ovatus, Faecalibacterium prausnitzii, Roseburia faecalis, Roseburia intestinalis, Eubacterium rectale* and *Ruminococcus torques*, and increased proportions of *Raoultella sp*. and *of E. coli*, suggesting a negative shift in the microbial community that favors the growth of pathogenic bacteria (65).

### Titanium Dioxide (TiO_2_) NPs

MET-1 model was also utilized in a custom colon reactor to determine the impact of TiO_2_NPs on the human microbiota. I*n vitro* studies reported a slight decrease in *B. ovatus* in favor of *Clostridium cocleatum*, after 48 h of treatment. However, longer exposure times (days) give significant changes in bacterial metabolites observed in the human colon microbiome, including in SCFAs production (66). In *vivo* studies performed in rodents proved very limited systemic absorption of TiO_2_NPs, therefore relevant dose for humans (2.5 mg/kg bw/d) reveal no significant microbiota changes in mice after 1 week of exposure (67). However, increasing the exposure time to 1 month and the NPs concentration to 100 mg/kg bw/d, has translated to an increased proportion of potentially harmful Actinobacteria and Proteobacteria and a decrease in the abundance of beneficial *Firmicutes* and *Bacteroidetes* in mice (68).

TiO_2_NPs administered in drinking water for 3 weeks at concentrations of 0, 2, 10, or 50 mg TiO_2_/kg BW/day revealed significant microbiota modulatory effects in a dose dependent manner. Proportions of *Lactobacillus* and *Allobaculum* were significantly elevated at all tested doses, while Parabacteroides were significantly elevated in mice treated with 50 mg TiO_2_/kg BW/day. Higher TiO_2_ concentrations (of 10 and 50 mg TiO_2_/kg BW/day) also decreased the proportion of *Adlercreutzia* and Unclassified *Clostridiaceae*, as compared to the untreated mice group. Moreover, co-occurrence analysis by examining microbial interactions from mice treated with various amounts of TiO_2_NPs revealed that certain genera (such as *Ruminococcus, Desulfovibrio*, and *Oscillospira*) are consistently associated with each other regardless of TiO_2_ treatment. Increased TiO_2_NPs intake (i.e. at the dose of 10 and 50 mg/kg BW/day) resulted in more significant connections within the network, as well as increased number of genera with significant contributions. Authors report that, while *Akkermansia* was not significantly involved in the microbial network of mice administered 0, 2, or 10 mg TiO2/kg BW/day, it is involved at a dose of 50 mg/kg involving numerous co-exclusion relationships (69). This recent evidence suggest that TiO_2_NPs could impair gut homeostasis which may in turn prime the host for disease development.

### Zinc Oxide (ZnO) NPs

ZnO is currently listed as a *generally recognized as safe* (GRAS) material by the *Food and Drug Administration* (USA) and is commonly used as food supplement or in food packaging. However, some *in vivo* studies report that ingested ZnO NPs could alter the intestinal microbiota and inflammation response. Shen and coworkers revealed that dietary exposure to coated ZnO in piglets results in a significant improvement in intestinal morphology and immunity, including increased villi length, elevated immunoglobulin A (IgA) levels, increased gene expression of IGF-1, occluding, zonula occludens 1, IL-10 and transforming growth factor β1 (TGF-β1), as well as reduced gut microbiota diversity (70). Studies made in piglets feed with 600 mg ZnO NPs/kg revealed an increase in the bacterial microbiota richness and diversity in ileum, while they decrease in cecum and colon. The relative abundances of *Streptococcus* in ileum, and *Lactobacillus* in colon were increased, while the relative abundances of *Lactobacillus* in ileum, *Oscillospira* and *Prevotella* in colon were decreased. These microbiota changes were ed with ZnO induced diarrhea in piglets, the effects of ingested 600 mg ZnO NPs/kg being similar to the ingestion of high dose of traditional ZnO (2,000 mg Zn/kg) (71).

Feng et al. investigated the impact of ZnO NPs on gut microbiota in ileal digesta and its relationship with blood metabolites in hens. Various concentrations of ZnO NPs, ranging 25, 50, and 100 mg/kg, were fed to hens for 9 weeks. Their study revealed that the bacterial community richness was negatively correlated with increasing amounts of ZnO NPs. The microbiota diversity was considerably reduced at ZnO NPs concentration of 100 mg/kg. Specifically, the proportions of Proteobacteria, Bacilli, and Fusobacteria were altered, while the amount of *Lactobacillus* was decreased. Moreover, microbiota modulation was correlated with changes in the plasma metabolism; for instance, methionine, lactate, and choline levels are positively associated with microbiota richness. These results suggest that ZnO NPs indirectly regulate blood metabolism *via* gut microbiota modifications (58). Dietary exposure of ZnO NPs was studied also on fish intestinal microbiota. Administration of 500 mg/kg ZnO NPs to *Cyprinus carpio* for 6 weeks indicated no significant difference in the gut microbiota of treated and untreated fish (61, 72).

### Silicon Dioxide (SiO_2_) NPs

Similar to ZnO, SiO_2_ NPs are intensively used food-grade particles, being approved by the European Union to serve as a food additive. SiO_2_ acts as anti-caking agent and carrier for flavors or fragrances in food; however, it may cause adverse effects in humans, such as silicosis (an occupational pulmonary disorder induced by inhalation of many free SiO_2_ particles) (73). Microbiota interactions were also observed *in vivo* after SiO_2_ ingestion. SiO_2_ NPs ingestion for 28 days determine disfunction of murine intestinal barrier, which might occur as a result of disordered gut microbiota (74). Significant decrease of intestinal bacterial phyla Verrucomicrobia, and bacterial genus *Akkermansia*, were observed after exposure. These bacteria are closely associated with the mucosal barrier and inflammatory responses in the intestine (72). Alterations in the gut microbiota composition were observed in patients who suffered from silicosis. The alteration of gut microbiota in these patients could be explained by the important amounts of inhaled SiO_2_ NPs reaching the gastrointestinal tract. The results showed that the levels of Actinobacteria and Firmicutes in patients with silicosis were lower than those in healthy subjects. In addition, lower levels of *Alloprevotella, Clostridiales, Devosia*, and *Rikenellaceae*_RC9, as well as higher *Lachnoclostridium* and *Lachnospiraceae* levels, were highlighted in silicosis patients (75). Mesoporous silica nanomaterials were continuously administered for 14 days by gavage in a mouse model. The exposure to silica nanomaterials resulted in significant damage to the intestinal mucosal epithelial structure, microbiota imbalance and enhanced intestinal inflammation, ed with oxidative stress and apoptosis of intestinal cells (76). Specifically, the gut microbiota diversity was obviously changed, especially the harmful bacteria. Intriguingly, the administration of SiO_2_ NPs dose similar to estimated human dietary intake (2.5 mg/kg bw/day) for seven consecutive days in mice increased gut microbial species richness as well as diversity. Researchers observed that the abundance of population belonging to *Lactobacillus sp* was correlated with the significantly increased levels of pro-inflammatory cytokines in the colon, including TNF-α, IL-1β, and IL-6. Nevertheless, no overall toxicity was observed in the mice exposed to SiO_2_ NPs at this dose (67).

Collectively, when analyzing the results of the above examples regarding the evaluated inorganic NPs, we could conclude that, although a few studies indicate they have no influence on gut microbiota or do not cause histological lesions, they mostly occur in the case of low doses. However, most of the reports with higher doses and long-term exposure, such as occupational exposure, still show that negative bio-effects can be found when inorganic NPs meet gut microbiota, particularly inflammation, diversity reduction and phyla imbalance (72). These effects are usually associated with inorganic NPs–derived free radicals that cause oxidative damage, but also direct contact to the microbiota bacteria cells, causing membrane damage or cell lysis caused by NPs agglomeration.

## Potential Mechanisms in EOs–NPs–Microbiota Triad

Despite their direct interaction with gut microbiota, NPs could also be utilized as vectors or shuttles for various drugs, bioactive molecules and mixtures. Inorganic NPs were investigated for their ability to stabilize and deliver plant extracts and compounds, including fruit EOs. Such nanostructured systems containing natural bioactive substances are widely investigated for their antimicrobial effect. Since EOs and NPs, separately, could impact on the gut microorganisms, the influence of such Eos–NPs systems in microbiota could be synergically enhanced.

When it comes to protect and control the delivery of EOs, numerous studies report the design of polymeric and lipidic nano- /micro- capsules, with wide applications in food industry and pharmacology (77). They may show improved antimicrobial effect, as compared to the plain EO administration, maintaining a longer time of activity, reduced toxicity and higher biodisponibility, which has been demonstrated on multiple bacterial pathogens (78, 79). The enhanced antimicrobial effect of EOs incapsulated in organic nano- and micro-capsules is usually explained by the better protection of the volatile EOs bioactive compounds, their controlled release and targeted delivery (80, 81). However, some polymers utilized to design efficient capsules for EOs, such as chitosan, showed intrinsic antimicrobial properties which could act synergically with the encapsulated EOs (82). Moreover, chitosan nanoparticles (CNPs) showed gut microbiota interference *in vivo* in weaned pigs. Diets supplemented with 0, 100, 200, and 400 mg/kg CNP fed for 28 days impacted on growth performance, immune status, microbiota composition and immune responses after LPS challenge in the weaned pigs. When a concentration of 400 mg/kg CNP was administered, results showed a decreased abundance of Clostridiaceae and increased abundance of Prevotellaceae and *Ruminococcaceae* families (83).

Due to their bioactivity and superior frequency in daily routine and environment, inorganic NPs have been increasingly gaining researcher's attention when designing EO-based nanosystems. Ag, Au, CuO, TiO_2_, SiO_2_, Fe_3_O_4_, and ZnO nanoparticles have been combined with EOs to obtain improved materials with antimicrobial properties (84). Such NPs-EOs systems have been combined often with organic nanomaterials to offer wider applications and ensure tunable properties.

Food industry has greatly benefited by the combination of inorganic NPs and EOs, since numerous advanced packaging materials were developed (85, 86). Inorganic NPs and EOs contained in such food and beverage packaging could easily go into the alimentary content and thus be ingested, reaching gut microbiota. Moreover, the nanomodified packaging would be disposed after use and reach environmental microbiota, if not adequately recycled. Although synergistic interactions of NPs and EOs are not yet investigated in gut microbiota, such nanostructured functional combinations demonstrated various antibacterial mechanisms against numerous food pathogens, but also against some gastrointestinal pathogens. Table 2 shows the main antimicrobial mechanisms in nanosystems containing inorganic NPs and EOs.

**Table 2:** Antimicrobial activity of NPs-EOs combinations in gut or food pathogens.

**Nanosystem**	**Main antimicrobial mechanism**	**Observed phenotype**	**Target microbial species**	**References**
ZnO NPs and *Opuntia humifusa* EO	Cell wall disruption (greater efficiency in gram positive bacteria)	Growth inhibition	*Bacillus cereus, Staphylococcus aureus, E. coli, Shigella sp., Pseudomonas sp*.	(87)
ZnO NPs and walnut extract	Cell wall disruption	Growth inhibition, bacteria killing	*S. aureus* and *E. coli*	(88)
Ag NPs and *Acacia rigidula* stem and root extract	Cell wall disruption, generation of reactive oxygen species (ros)	Growth inhibition, bacteria killing	*Bacillus subtilis, E. coli, Pseudomonas aeruginosa*,	(89)
Ag NPs and *Capparis zeylanica* extract	Intracellular protein and dna disruption	Growth inhibition, antimicrobial	*Staphylococcus epidermidis, Enterococcus faecalis, Salmonella paratyphi, Shigella dysenteriae, Candida albicans*	(90)
Ag NPs, *Picea abies* and *Pinus nigra* bark EO	Generation of ros (greater efficiency in gram negative bacteria)	Growth inhibition, antimicrobial	Methicilin susceptible and resistant *S.aureus* (MSSA, MRSA), *S. epidermidis, Streptococcus pyogenes, E. coli, P. aeruginosa* and *C. albicans*	(91)
Ag NPs and *Butea monosperma* bark extract	Generation of ros	Growth inhibition, antimicrobial	*S. aureus, E. coli* and *Aspergillus niger*	(92)
Ag NPs and *Cymbopogon citratus EO*	Generation of ros	Growth inhibition	*B. cereus, E. coli, Salmonella paratyphi, Vibrio cholera* and *Shigella flexneri*	(93)
Ag NPs and *Agastache foeniculum* extracts	Unspecified	Antibacterial	*S. aureus, S. haemolyticus, Streptococcus pneumoniae, Klebsiella pneumoniae, Acinetobacter baumannii*	(94)
Au NPs and *Annona muricata* EO	Possible cell wall disruption	Antimicrobial	*S. aureus, Enterococcus faecalis, Clostridium sporogenes, K. pneumoniae, C. albicans, Aspergillus flavus, Fusarium oxysperium, Penicillium camemeri*	(95)
Ti*O*_2_ NPs and *Aloe vera* leaf extract	Generation of ros	Growth inhibition, antimicrobial	*S. aureus, E. coli, C. albicans, A. niger*	(96)
Ti*O*_2_ NPs and *Garcinia zeylanica* EO	Generation of ros	Growth inhibition, bacterial dose specific killing	MRSA	(97)
CuO NPs, *Vernonia amygdalina* and *Amaranthus hybridus* plant extracts	Possible cell wall disruption	Growth inhibition	*S. aureus, B. subtilis, B. megaterium, E. coli, P. aeruginosa, Serratia marcecens, C. albicans, A. niger, Penicillium chrysogenum*	(98)
CuO NPs and *Catha edulis* leaf extract	Possible cell wall disruption (greater efficiency on gram negative bacteria)	Growth inhibition, bacterial dose specific killing	*S. aureus, Streptococcus pyogenes, K. pneumoniae, E. coli*	(99)
CuO NPs and *Bergenia ciliata* extract	Ions release, oxidative stress	Growth inhibition	*S. aureus, B. subtilis, E. coli, Salmonella typhi*	(100)
CuO NPs and *Abutilon indicum* extract	Ros generation	Growth inhibition	*S. aureus, B. subtilis, E. coli, Klebsiella sp*.	(101)
*Fe*_3_*O*_4_ NPs and *Lagenaria siceraria* EO	Ros generation	Growth inhibition	*Staphylococcus aureus* and *E. coli*	(102)
*Fe*_2_*O*_3_ NPs and *Carica papaya* extract	Ros generation	Growth inhibition	*S. aureus, Klebsiella* sp., *E. coli, Pseudomonas* sp.	(103)
FeO NPs and *Ruellia tuberosa* EO	Ros generation	Growth inhibition	*S. aureus, E. coli, Klebsiella pneumoniae*	(104)

The most probable interactions of NPs and EOs in the gut could be related to their fate in the gut. EOs and NPs are absorbed and eliminated with different kinetics in the gastrointestinal tract, but both modulate the same key processes, such as: gut inflammation (105, 106), local oxidative stress (35, 107), metabolites synthesis (i.e., SCFAs) (22, 107), growth and development of bacterial pathogens and microbiota members (58, 107), and, ultimately, can induce *in situ* toxicity of host and microbial cells, in a dose and exposure time dependent manner (72). In this respect, synergistic and antagonist interactions should be considered when investigating microbiota modulation derived by NPs and EOs.

**Figure 1:**
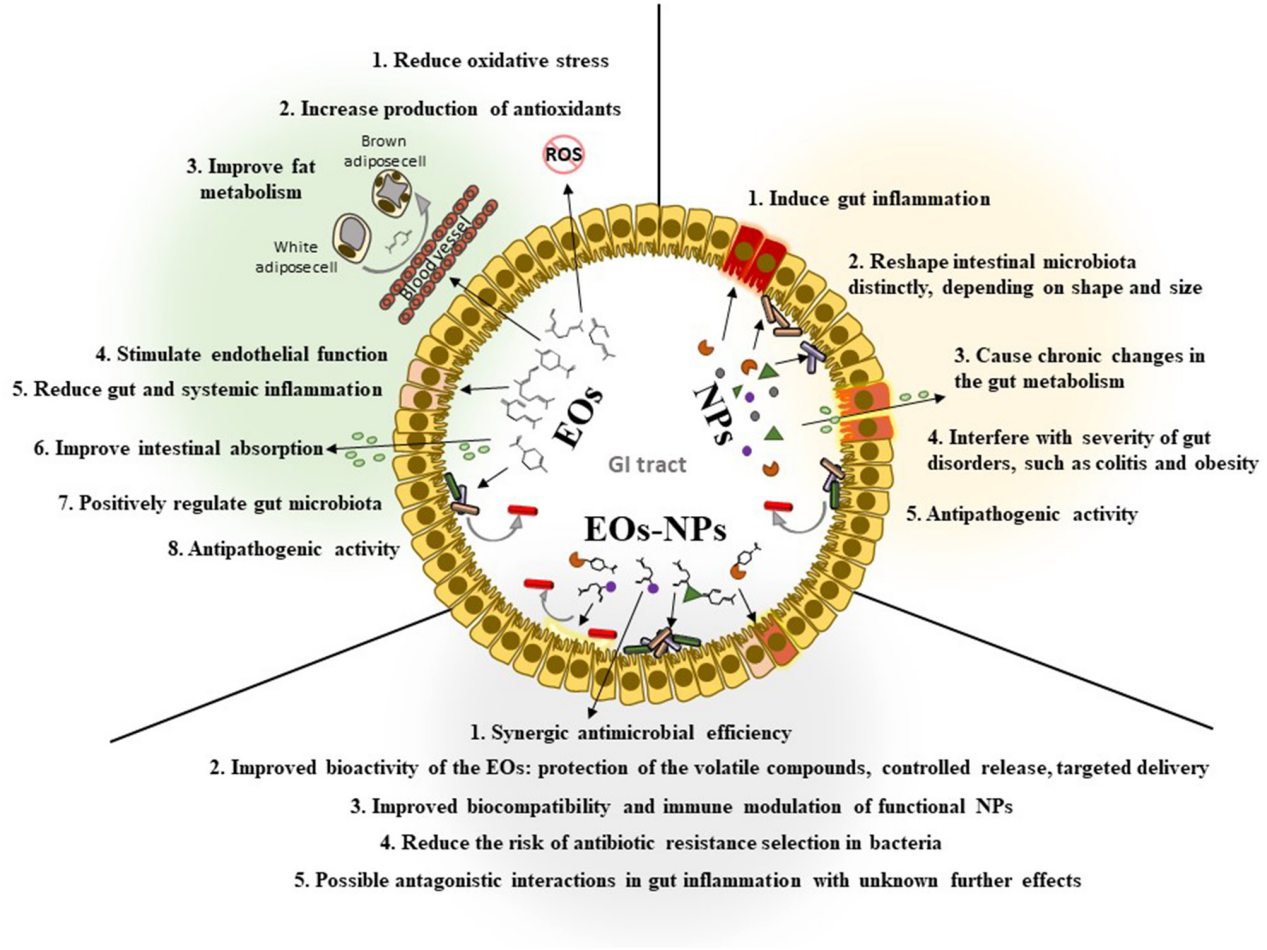
Scheme revealing the main mechanisms which may be modulated by EOs, NPs, and their association in the gut.

However, very diverse interferences were described separately for inorganic NPs and EOs, and these differences could lead to more complex and difficult to elucidate interactions in the NPs-EOs-microbiota triad. Figure 1 highlights the main processes which could be modulated in the gut by EOs and NPs.

### Gut Inflammation

EOs are recognized for their wide anti-inflammatory activity. This corelates with their significant reduction of relevant proinflammatory cytokines levels, such as necrosis factor alpha (TNFα), IL-1α, IL-1β, IL-6, IL-13, and NO (108, 109); as well as induction of anti-inflammatory markers, such as: IL-5, IL-13, expression of HO-1 (Nrf2-HO-1 pathway), increased production of CD8^+^, CD16^+^ cells, and IgA by activation of anti-inflammatory M2 phenotype of macrophages (110, 111).

On the other hand, inorganic NPs usually induce gut inflammation of various intensity, from mild to severe inflammatory processes, depending on the NPs type, exposure time and concentration (112). They usually increase the production of pro-inflammatory molecules, TNF-α, interferon-gamma (IFN-γ), IL-12 and of the Th2 cytokine IL-4 in the intestine (53, 113). These are opposite effects of EOs and NPs in gut inflammation and could lead to antagonistic interactions with unknown manifestation on the gut microbiota. Numerous studies demonstrate particular associations of some microbiota phyla with inflammatory processes in the gut (114, 115), however, researchers are still arguing if microbiota composition may induce gut inflammation, or inflammatory factors found in high amounts in the gut determine the modulation of microbiota composition.

### Microbiota Diversity

Microbiota composition and diversity is modulated distinctly by EOs and NPs; while the first tend to reduce the occurrence of pro-inflammatory phyla (37), the latest show non-concluding results regarding specific NPs induced microbiota modulation toward the pro-inflammatory phyla (53). However, the currently available literature reveal that NPs exhibit a moderate to extensive impact on intestinal microbiota composition, highlighting a recurrent signature that favors gut colonization by pathobionts at the expense of beneficial bacterial strains. Such particular changes are frequently observed in metabolic and inflammatory conditions, for instance in inflammatory bowel diseases (IBD, which include Crohn's disease and ulcerative colitis), obesity and colorectal cancer (53, 116), which are highly influenced by the microbiota. Generally, the inorganic NPs-induced gut microbiota signature resembles that of dysbiosis-associated human diseases, characterized by alteration of the Firmicutes/ Bacteroidetes ratio together with depletion of *Lactobacillus* and enrichment of Proteobacteria (53, 60, 70, 117). Moreover, microbe-microbe interactions could also impact the microbiota-EOs-NPs triad. This idea is supported by a recent study demonstrating relevant microbiome modulation based on probiotic application. Moreover, along with their impact in microbiota diversity changes, probiotics are also studied as potent metabolic modulators, being proposed for the management of health conditions, such as obesity and cancer (118).

### Anti-pathogenic Activity

Both EOs and inorganic NPs showed unquestionable anti-pathogenic effects, expressed against numerous microorganisms, including gut and food pathogens. Also, synergistic interactions and enhanced antibacterial activity was frequently reported in recent studies when utilizing EOs-NPs combinations both *in vitro* and *in vivo*. It seems that, generally, EOs could target microbial pathogens (119), while protecting beneficial bacteria (i.e., supporting the growth of anti-inflammatory microbiota phyla), NPs seem to exert undifferentiated antimicrobial activity, impacting similarly in commensal and pathogenic microorganisms (120). Since some antimicrobial mechanisms of EOs and NPs are similar and show unspecific targets among different bacteria (i.e., oxidative stress in the bacterial cells, membrane damage, permeability changes), it is difficult to explain their potential different effects in antimicrobial effect against microbiota or pathogenic strains. However, impressive research developed in recent years propose innovative solutions to target specific microorganisms (i.e., pathogenic species, virulent or resistant bacterial strains) by using tailored NPs (121). Such nanosystems could act as delivery agents for EOs, ensuring their targeted effect against intended pathogens (122). Moreover, NPs-EOs combinations could make a significant impact in antibiotic resistant bacteria by: i) targeting multiresistant strains and ii) interfering with QS signaling, virulence genes expression and progress of the infectious process and iii) reducing the risk of selection for resistant mutants, which would be difficult to escape the antimicrobial mechanisms imposed by two agents in the same time.

### Short-Chain Fatty Acids (SCFAs)

Gut microbiota regulates intestinal mucosa permeability, nutrient uptake and is involved in digestion through the production of molecules with fermentative, saccharolytic (with the production of SCFAs) (123) and proteolytic properties. It is widely recognized that microbiota SCFAs production is highly altered by EOs and NPs, they being the most studied microbial metabolites.

SCFAs are monocarboxylic acids with maximum six carbons atoms, being the result of the microbial anaerobic fermentation process of indigestible carbohydrates such as dietary fibers and resistant starch, in the colon (124). Major SCFAs in GI are acetate, propionate, and butyrate (>95%), the quantity being dependent on the diet and microbiota pattern (125). These main three SCFAs are found normally in molar ratios varying from 3:1:1 to 10:2:1. Most SCFAs are metabolized to CO_2_. Butyrate is used locally as an energy source for colonocytes, caliciform cells, or Paneth cells and regulates apoptosis, cell differentiation, and changes in the chemical structure of proteins and nucleic acids. In contrast, acetate and propionate pass into circulation, from where they are taken up by the liver and peripheral organs, where they are used as substrate for gluconeogenesis or lipogenesis (27, 126). Acetate and propionate are mainly produced by *Bacteroidetes*, while butyrate is produced mostly by *Firmicutes* (21).

Most SCFAs are absorbed in the proximal colon in exchange for bicarbonate, which neutralizes the luminal pH. As a result, the pH in the cecum is lower (5.5) than the rectum (6.5). This phenomenon can be explained by the lower capacity of *Bacteroides* compared to *Firmicutes* species to tolerate the presence of SCFAs at pH 5.5 than at pH 6.5 resulting in a microbial composition shift. This shift limits propionate and stimulates butyrate production (116).

All gut commensals ferment pyruvate for butyrate production, whereas the pathogenic bacteria, for example *Fusobacterium*, utilizes different pathways like those for Glutamate (4-aminobutyrate) and Lysine, which are associated with a release of harmful byproducts like ammonia (**85**).

Reduced levels of commensal bacteria in dysbiosis, such as butyrate producers, and the consequent ecological gap, could promote the growth of some intestinal opportunistic pathogens (127). It was also observed an inverse correlation between the abundance of *Faecalibacterium prausnitzii*, a major butyrate producer, and the local and systemic inflammation (8). A decreased production of SCFAs is correlated with the extinction of the microbial taxa producing the glycoside hydrolases as a consequence of a decreased dietary fibers intake (128).

SCFAs could be also absorbed and transported into the portal circulation, being used as energy source by hepatocytes (129). SCFAs are now considered as potential therapeutics (116), having the ability to improve the gut health, contributing to intestinal barrier integrity, mucus production, protection against inflammation and colorectal cancer (4, 130). The influence of SCFAs modulation on the central nervous system (CNS) have lately attracted the attention of researchers, being a subject of intense debate in recent years.

### Distal Effects

Dietary changes, infections, but mostly antibiotherapy drastically act on the gut microbiota composition, leading to frequent opportunistic infections, but also long-term impact on brain and behavior (130, 131). The ability of NPs and EOs to modulate microbiota diversity could also lead to distal effects in the host, impacting on various organs and tissues. The connection between the microbiome, gut and brain is a new concept called *microbiome-gut-brain axis* (131–133). So, there is a real crosstalk between the gut microbiota and brain, with some intestinal bacteria able to modulate neurobehavioral responses, by direct effects of the bacterial cells, their metabolites that mimic some host molecules, such as neurotransmitters, and/or stimulation of host inflammation (62).

Despite their demonstrated antimicrobial activity and the plethora of NP applications as additives in various products, some studies are reporting long-term and long-distance noxious effects, depending on the route of pentetration into the body, such as dysbiotic and metabolic alterations, mucosal immunity modifications, and neurotoxicity (62, 134).

It seems that AgNPs can accumulate in the GI tract, thereafter enter in the blood and can reach the brain, being able to cross the blood–brain barrier (BBB) or *via* retrograde axonal transmission (135).

A short-term experimental study using radioactive NPs, has confirmed that maternal oral exposure to AgNPs of ~ 50 nm (polyvinyl pyrrolidine coating) leads to placental and milk transfer and subsequent accumulation in the neonate brains (136).

Although it is unclear how NPs and EO could affect gut microbiota, it is becoming obvious that some disorders in gut bacteria can affect host health outside the gut, especially neural development and consequent risk for neurobehavioral disorders, such as autism spectrum disorders (ASD). It seems that over-abundance of a *Ruminococcus spp*. occurs in human patients with generalized anxiety disorder (137).

Among the effects observed in animals developmentally exposed to NPs, the most concerning is the reduced abundance of beneficial bacteria *Bifidobacterium spp*., known for their probiotic properties. One study showed that supplementation with a probiotic containing *Bifidobacterium spp*. led to improved health status in children with ASD and gastrointestinal disorders (2). On the contrary, reductions of the bifidobacteria number may induce neurobehavioral and GI diseases in rodent models and susceptible humans too (62).

Another effect is the inverse association between abundant *Prevotella spp*. and steroid hormone biosynthesis. Steroid hormones, including testosterone and aromatization of testosterone to estrogens, is essential in reproduction, but also in regulation of many neural functions. Reductions in these steroid hormones, especially estrogens, is linked to later neurobehavioral disorders, such as Alzheimer's Disease (AD) (62, 138, 139). The increased abundance of *Prevotella spp. and Ruminococcus spp*. also disturbs the biosynthesis pathway of unsaturated fatty acids. These fatty acids, especially docosahexaenoic acid (DHA), are essential for normal cognitive functions such as learning and memory, stress management, regulating emotional responses, inhibiting inflammation in the central nervous system, reducing the impulsive behavior, AD, and other brain disorders (140–142). SCFAs are considered key candidate mediators of gut-brain communication, and altered SCFA production has been demonstrated in a variety of neuropathologies (130, 143, 144).

## Concluding Remarks

Microbiota is still a widely unexplored resource in understanding human physiology and numerous pathological conditions. This microbial community residing mostly in the gut is constantly exposed to external factors, especially diet, but also antibiotics and xenobiotic substances.

EOs are widely studied for their gastrointestinal interference, being potent modulators for gut inflammation, metabolic reactions and microbiota diversity. On the other hand, micro- and nano-particles are frequently reaching human microbiota, and they can also impact on key reactions in the gut. Although the intimate interactions between microbiota, EOs and NPs are not fully deciphered, these factors could show similar, synergic, or even antagonist effects, which could significantly impact on the host health. EOs are generally recognized as inflammation-reduction factors, while inorganic NPs usually promote inflammation in the gut after ingestion. However, both anti-inflammatory and pro-inflammatory mechanisms bring biochemical changes which might be responsible for the modulation of gut microbiota in terms of distribution, diversity and metabolic response.

EOs and plant-derived molecules have been empirically known and used in ethnomedicine, prophylaxis or immune stimulation, but recent science propose their use in various combinations with antibiotics, NPs, and other drugs which could show unpredicted effects, since their intimate mechanisms of action are not known.

This study offers new perspective in the investigation of antimicrobial effects of NPs and EOs, which are currently widely investigated alternatives to fight antibiotic resistant bacteria. Their antimicrobial efficiency should be also considered in terms of microbiota modulation capacity, in order to develop optimized therapeutics with lower side effects.

## Author Contributions

Conceptualization and supervision: VL and A-MH. Writing–original draft preparation: VL, A-MH, CC, and LD. Editing: CC and LD. Funding: A-MH. All authors have read and agreed the final version of this paper.

## Funding

This work was supported by a grant of the Ministry of Research, Innovation and Digitization, CNCS/CCCDI – UEFISCDI, project number 147TE/2020, within PNCDI III.

## Conflict of Interest

The authors declare that the research was conducted in the absence of any commercial or financial relationships that could be construed as a potential conflict of interest.

## Publisher's Note

All claims expressed in this article are solely those of the authors and do not necessarily represent those of their affiliated organizations, or those of the publisher, the editors and the reviewers. Any product that may be evaluated in this article, or claim that may be made by its manufacturer, is not guaranteed or endorsed by the publisher.
